# VtaA8 and VtaA9 from *Haemophilus parasuis* delay phagocytosis by alveolar macrophages

**DOI:** 10.1186/1297-9716-43-57

**Published:** 2012-07-27

**Authors:** Mar Costa-Hurtado, Maria Ballester, Nuria Galofré-Milà, Ayub Darji, Virginia Aragon

**Affiliations:** 1 Centre de Recerca en Sanitat Animal, (CReSA), UAB-IRTA, Campus de la Universitat Autònoma de Barcelona, 08193, Bellaterra, Barcelona, Spain; 2 Institut de Recerca i Tecnologia Agroalimentàries (IRTA), Barcelona, Spain

## Abstract

*Haemophilus parasuis,* a member of the family *Pasteurellaceae,* is a common inhabitant of the upper respiratory tract of healthy pigs and the etiological agent of Glässer’s disease. As other virulent *Pasteurellaceae*, *H. parasuis* can prevent phagocytosis, but the bacterial factors involved in this virulence mechanism are not known. In order to identify genes involved in phagocytosis resistance, we constructed a genomic library of the highly virulent reference strain Nagasaki and clones were selected by increased survival after incubation with porcine alveolar macrophages (PAM). Two clones containing two virulent-associated trimeric autotransporter (VtaA) genes, *vtaA8* and *vtaA9*, respectively, were selected by this method. A reduction in the interaction of the two clones with the macrophages was detected by flow cytometry. Monoclonal antibodies were produced and used to demonstrate the presence of these proteins on the bacterial surface of the corresponding clone, and on the *H. parasuis* phagocytosis-resistant strain PC4-6P. The effect of VtaA8 and VtaA9 in the trafficking of the bacteria through the endocytic pathway was examined by fluorescence microscopy and a delay was detected in the localization of the *vtaA8* and *vtaA9* clones in acidic compartments. These results are compatible with a partial inhibition of the routing of the bacteria via the degradative phagosome. Finally, antibodies against a common epitope in VtaA8 and VtaA9 were opsonic and promoted phagocytosis of the phagocytosis-resistant strain PC4-6P by PAM. Taken together, these results indicate that VtaA8 and VtaA9 are surface proteins that play a role in phagocytosis resistance of *H. parasuis.*

## Introduction

*Haemophilus parasuis* is a member of the family *Pasteurellaceae* and a common inhabitant of the upper respiratory tract of healthy pigs. It is also known as the etiological agent of Glässer’s disease in swine, a systemic disease characterized by fibrinous polyserosytis, which causes high morbidity and mortality in piglets. *H. parasuis* can also produce pneumonia and sudden death [1]. Glässer’s disease has gained considerable importance in recent years and it is recognized as one of the main causes of economic loss in the pig industry [2].

Little is known about the pathogenesis and the virulence factors of *H. parasuis*. Some putative virulence factors have been reported [3-8], including a family of trimeric autotransporters, designated virulence-associated trimeric autotansporters (VtaA) [9]. Trimeric autotransporters are present in Gram-negative bacteria and they have been widely confirmed as virulence factors in other bacteria [10,11].

Vahle et al. [12] determined the dynamics of infection with *H. parasuis* after intranasal inoculation with a systemic isolate, showing that *H. parasuis* has to survive host pulmonary defences in order to produce systemic disease. In the lung, the first line of defence is composed of alveolar macrophages, whose main role is the elimination of airborne pathogens and other environmental particles [13,14]. The phagocytosed particles are subsequently destroyed as they progress along the degradative endocytic pathway, culminating in the formation of the mature phagolysosome. [15]. Like other virulent *Pasteurellaceae*[16-19], *H. parasuis* has evolved mechanisms to prevent phagocytosis as part of its pathogenic profile, as demonstrated in a previous study [20]. These mechanisms allow microorganisms to avoid destruction via the degradative endocytic pathway and in some cases prevent phagocytosis [21].

In order to identify the genes involved in this virulence mechanism of *H. parasuis*, we constructed a genomic library of the highly virulent reference strain Nagasaki and clones from the library were selected by incubation with porcine alveolar macrophages (PAM). Two v*taA*, *vtaA8* and *vtaA9* were identified and their role in phagocytosis resistance was explored, demonstrating for the first time the involvement of these two proteins in resistance to phagocytosis in *H. parasuis*.

## Materials and methods

### Bacterial strains and plasmids

Bacterial strains and plasmids used in this study are listed in Table 1. *Escherichia coli* EPI300 was used as the host for recombinant plasmids and was grown on Luria-Bertani (LB) agar or in LB broth, supplemented with 100 μg/mL ampicillin, 12.5 μg/mL chloramphenicol (pCC1FOS) or 30 μg/mL chloramphenicol (pACYC184), as appropriate. *H. parasuis* strains were grown on chocolate agar.

**Table 1:** Bacterial strains and plasmids used in this study.

	**Description**	**Reference**
**STRAINS**		
***H. parasuis***		
Nagasaki	virulent reference strain, serovar 5	[22]
PC4-6P	virulent field strain,serovar 12	[20]
SW114	non-virulent reference strain, serovar 3	[22]
F9	non-virulent field strain, serovar 6	[20]
***E. coli***		
EPI300	Phage T1-resistant	Epicentre Biotechnologies
**PLASMIDS**		
pCC1FOS	inducible copy, CmR	Epicentre Biotechnologies
pACYC184	low copy, CmR, TetR	ATCC number 37033
pEGFP	*gfp*, AmR	Clontech
pCC1FOS-8	pCC1FOS with a 47,195 bp insert, including *vtaA8*	this paper
pCC1FOS-9	pCC1FOS with a 38,659 bp insert, including *vtaA9*	this paper
pMCH-vtaA8	*vtaA8* cloned in the BamHI site of pACYC184	this paper
pMCH-vtaA9	*vtaA9* cloned in the BamHI site of pACYC184	this paper

Cm: Chloramphenicol.

Tet: Tetracycline.

Am: Ampicillin.

### Genomic library production

A genomic library derived from the *H. parasuis* virulent strain Nagasaki was produced with the CopyControl^TM^ Fosmid Library Production kit (Epicentre Biotechnologies, Madison, USA) with pCC1FOS™, according to the manufacturer’s instructions. Genomic DNA from the Nagasaki strain was purified with a Nucleospin blood kit (Macherey-Nagel, Düren, Germany) and fragments of approximately 40 kb were used for library construction. The genomic library consisted of 300 fosmid clones, to ensure a complete library with a 99% probability.

### Sequencing, PCR and cloning

To identify the genomic sequence included in selected fosmids, these clones were induced to high copy number and pCC1/pEpiFOS forward and reverse primers (Epicentre Biotechnologies) were used in sequencing reactions using a BigDye Terminator v.3.1 kit and an ABI 3100 DNA sequencer (Applied Biosystems, Carlsbad, USA). The complete sequence included in each fosmid was deduced by comparison with the Nagasaki genome sequence [9].

In addition, two genes of interest, *vtaA8* and *vtaA9*, were PCR-amplified from the corresponding fosmid clones with primers GCGCGGATCCTCTTAGTTTTGTGTAACTCTT and GCGCGGATCCTTCTAATTTATAGGTGCTAGATTAC (BamHI site in primer sequence is underlined) and Accuprime^TM^ Taq DNA polymerase high fidelity (Invitrogen, Madrid, Spain). The amplicons were then digested with BamHI and cloned into the BamHI site of pACYC184 to yield pMCH-vtaA8 and pMCH-vtaA9 (Table 1) for further study.

### Phagocytosis assay

Phagocytosis assays were performed as described before [20]. Briefly, porcine alveolar macrophages (PAM) were seeded in 6-well plates at a concentration of 5 × 10^5^ cells in 3 mL per well of Dulbecco’s modified Eagle’s medium (DMEM) supplemented with 10% fetal bovine serum (FBS) and 1% L-glutamine, complete DMEM (CDMEM). Plates were incubated at 37°C with 5% CO_2,_ and after attachment of the cells to the wells (for a minimum of 1 h up to overnight incubation), the wells were inoculated with bacteria at a multiplicity of infection (MOI) of 200. Selected *E. coli* transformants were also transformed with pEGFP (plasmid carrying the green fluorescent protein [GFP] gene) for this assay, and fluorescein isothiocyanate (FITC)-labeled *H. parasuis* strains were used as controls. After incubation at 37°C for different times, wells were washed to eliminate unbound bacteria and PAM with associated bacteria were detected by flow cytometry in an EPICS XL-MCLTM flow cytometer (Beckman Coulter, Madrid, Spain). Assays were performed in duplicate and were repeated using PAM from different animals.

In some experiments, pMCH-vtaA8 and pMCH-vtaA9 were incubated in the same well with PAM to examine their interaction.

### Bacterial survival after incubation with macrophages

For survival studies, an MOI of 1 was used in the phagocytosis assay. After 1 h, 2 h, 3 h and 5 h PAM were lysed with 0.1% saponin and pippeting. Live bacteria in the wells were quantified by dilution and plating. Duplicate wells were used and the assay was repeated four times.

### Monoclonal antibody production

Monoclonal antibodies (mAb) were produced against VtaA8 and VtaA9 by immunizing BALB/c mice with their recombinant passenger domains. All procedures involving animals were performed in accordance with the regulations required by the Ethics Commission in Animal Experimentation of the Generalitat de Catalunya (Approved Protocol Number 5767). Passenger domains of VtaA8 and 9 were produced and purified as recombinant proteins (rVtaA8 and rVtaA9) following the protocol of Olvera et al. [23].

Mice were subcutaneously immunized with 50 μg of purified rVtaA8 or rVtaA9 with complete Freund’s adjuvant, followed by a second dose of protein with incomplete Freund’s adjuvant 2 weeks later. Two weeks after the second immunization and one day before the sacrifice, animals were boosted with 10 μg of protein in saline solution. Hybridomas were produced by fusion of lymphoid cells with X63AG8 myeloma cells following standard methods [24]. Undiluted supernatants from growing hybridomas were screened by indirect ELISA, using 100 ng/well of rVtaA8 or rVtaA9. Positive hybridomas were sub-cloned and further characterized by western blotting after purification using a protein A-Sepharose column (GE Healthcare, Barcelona, Spain). Western blotting was performed following standard methods in an SDS-PAGE system with 10% polyacrylamide gels and nitrocellulose membranes [25].

Isotyping of selected mAb (69C6, 80H8 and 95F4) was performed with a mouse monoclonal antibody isotyping test kit (AbD Serotec, Kidlington, UK) according to the manufacturer’s instructions.

### Bacterial VtaA8 and VtaA9 localization

MAb were used to detect VtaA8 and VtaA9 on the bacterial surface by flow cytometry. EPI300 (pACYC184), (pMCH-vtaA8) and (pMCH-vtaA9) were resuspended to an OD_660_ of 1. Monoclonal antibodies were used at 50 ng/μL, and incubated with the bacterial suspensions overnight on ice. After 3 washes with 1% bovine serum albumin (BSA) in phosphate buffered saline (PBS) to eliminate unbound antibodies, samples were incubated with a FITC-conjugated goat anti-mouse IgG (Jackson ImmunoResearch Europe Ltd, Newmarket, UK). After 2 washes to eliminate unbound conjugate, bacterial suspensions were analysed by flow cytometry in an EPICS XL-MCLTM flow cytometer. *H. parasuis* PC4-6P was also processed in the same manner, with strain F9 as the negative control.

### Opsonophagocytosis

The opsonic capacity of antibodies against VtaA8 and VtaA9 was tested in an opsonophagocytosis assay. Phagocytosis-resistant strain PC4-6P was used in these assays and was opsonized with monoclonal antibodies. A hyperimmune rabbit serum produced against the Nagasaki strain was used as a positive control. Opsonization of PC4-6P was performed overnight on ice using 200 ng/μL of each monoclonal antibody. After washes to eliminate unbound antibodies, bacterial suspensions were labeled with FITC and used in phagocytosis assays in triplicate wells as described above. F9 and SW114 were also included as control of phagocytosis.

### Immunofluorescence

Bacterial intracellular localization was determined by immunofluorescence following a previously published protocol with modifications [26]. PAM were seeded on glass coverslips in 6-well plates with CDMEM and phagocytosis assays were performed as described above. Early endosomes were detected with antibodies against early endosome antigen 1 (EEA1), purchased from Santa Cruz Biotechnologies (Santa Cruz, USA) and acidic compartments were detected with Lysotracker Red DND-99 (Invitrogen). *H. parasuis* strains PC4-6P (virulent) and F9 (non-virulent) were used to examine intracellular localization after 1 h of incubation at 37°C with PAM. For *E. coli* clones (pACYC184, pMCH-vtaA8 and pMCH-vtaA9; all with pEGFP), 1 h of pre-incubation on ice was performed to allow bacterial attachment to macrophages. Then, plates were incubated for 30 min and 1 h at 37°C. For the detection of acidic compartments, Lysotracker was added to the wells at a 1:2000 dilution at the same time as the bacterial inoculum. After the corresponding incubation (30 min or 1 h), coverslips were washed with PBS and immediately fixed with 4% paraformaldehyde (PFA) in PBS for 15 min at room temperature (RT). After fixing, samples were permeabilized with 0.5% Triton X-100 for 15 min at RT. Samples were then blocked with donkey serum (Jackson ImmunoResearch) for 1 h at RT and then incubated at 4°C overnight with goat anti-EEA1 diluted 1:20 in 3% BSA-PBS. *H. parasuis* strains were detected inside the PAM with a 1:100 dilution of a mix of hyperimmune rabbit sera produced against Nagasaki and SW114 strains. After three washes with PBS, coverslips were incubated with Cy3-conjugated donkey anti-goat IgG (H + L) or FITC-conjugated anti-rabbit IgG (whole molecule) for 1 h in 3% BSA-PBS at RT. *E. coli* clones were detected by GFP expression. Finally, nuclei were counterstained with 4',6-diamidino-2-phenylindole (DAPI) at 1 μg/mL and coverslips were mounted with Vectashield. Fluorescent images were viewed on a Nikon eclipse 90i epifluorescence microscope equipped with a DXM 1200F camera (Nikon Corporation, Japan). Image stacks were captured using OLYMPUS FluoView FV1000 confocal microscope (×60/NA 1.35 objective). Z stack images were acquired at intervals of 1 μm. Images were processed using FV10-ASW 1.7 Viewer software from Olympus and Image J v1.46d software [27].

To determine the percentage of bacteria that co-localized with each marker, approximately 100 cells were analyzed in each experiment. The results were calculated from 2 independent experiments as the percentage of cells with bacteria co-localizing with each marker within the group of infected PAM and statistical differences were determined by a Chi-squared test using a significance threshold of *p* < 0.05.

### Apoptosis assay

Apoptosis was detected by immunofluorescence assay using caspase-3 antibodies (Asp175; Cell Signaling technology, Danvers, USA). Apoptotic cells reacting with the antibody were observed after 1 h at RT incubation with a DyLight 549 goat anti-rabbit IgG (H + L) (Jackson ImmunoResearch) under a fluorescence microscope.

## Results

### Selection of phagocytosis-resistant clones

In order to identify genes involved in phagocytosis resistance, a genomic library derived from the Nagasaki strain was screened by sequential incubations with PAM. Several clones were selected based on increased survival as compared to *E. coli* EPI300 (pCC1FOS).

Twenty clones were partially sequenced and their complete sequences were deduced by comparison with the Nagasaki genomic sequence. Two of those fosmid clones (pCC1FOS-8 and pCC1FOS-9) contained genes encoding 2 different trimeric autotransporter genes (*vtaA8* and *vtaA9*, respectively) and were selected for further study (Table 1). Insert size was 47,195 bp in pCC1FOS-8 (corresponding to nucleotides 912,650 to 959,845 of the SH0165 genome; accession number NC_011852) and 38,659 bp in pCC1FOS-9 (corresponding to nucleotides 676,395 to 700,323 of the SH0165 genome).

**Figure 1:**
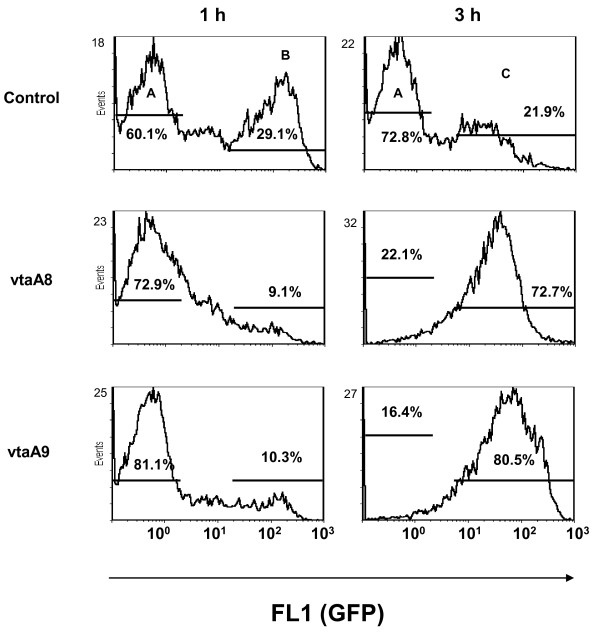
**VtaA8 and VtaA9 reduced the bacterial interaction with porcine alveolar macrophages (PAM).*** E. coli* EPI300 clones pMCH-vtaA8 (vtaA8) and pMCH-vtaA9 (vtaA9), also carrying pEGFP, were incubated for 1 h or 3 h with PAM and the interaction with the macrophages was analysed by flow cytometry. Results show the macrophages with fluorescent bacteria given by GFP and measured in FL1 (gates B and C, for 1 h and 3 h, respectively). *E. coli* with the empty vector pACYC184 and pEGFP was used as a control. Percentages of macrophages in gates A, B and C are included in the panels.

*E. coli* EPI300 (pCC1FOS-8) and EPI300 (pCC1FOS-9) were transformed with pEGFP and analysed in phagocytosis assays by flow cytometry. Both clones showed a reduced interaction with PAM as compared to *E.coli* EPI300 (pCC1FOS pEGFP) (not shown). A randomly selected fosmid clone was used as the control and it showed the same interaction with PAM as the control strain carrying the empty vector (data not shown). To determine the role of *vtaA8* and *vtaA9*, the genes were PCR-amplified and cloned into pACYC184 to give pMCH-vtaA8 and pMCH-vtaA9, and were introduced into *E. coli* EPI300 together with pEGFP. Compared to EPI300 (pACYC184 pEGFP), EPI300 (pMCH-vtaA8 pEGFP) and EPI300 (pMCH-vtaA9 pEGFP) showed a reduced interaction with PAM in phagocytosis assays (Figure 1). After 1 h of incubation at 37°C, 29% of PAM incubated with EPI300 (pACYC184 pEGFP) had associated bacteria (gate B of control panel) and at 3 h almost all bacteria were degraded, as shown by a reduction in fluorescence intensity (xmean of 40, gate C of control panel). In contrast, PAM incubated 1 h with EPI300 (pMCH-vtaA8 pEGFP) or EPI300 (pMCH-vtaA9 pEGFP) showed a lower percentage of macrophages with fluorescent bacteria than the control with the empty vector (around 10%; Figure 1, gate B of panels vtaA8 and vtaA9). After 3 h of incubation, a high percentage of PAM had associated EPI300 (pMCH-vtaA8 pEGFP) and EPI300 (pMCH-vtaA9 pEGFP) (Figure 1, gate C of panels vtaA8 and vtaA9). However, the mean fluorescence intensity of these macrophages was higher than the macrophages with control bacteria (gate C xmean of 50 and 95.5, for vtaA8 and vtaA9, respectively), indicating that those clones required longer periods of incubation before finally being phagocyted and for complete bacterial destruction. Simultaneous incubation of both the clones had no synergic effect in phagocytosis.

As the control, growth curves of EPI300 (pACYC184), (pMCH-vtaA8) and (pMCH-vtaA9) were evaluated and no differences were observed (data not shown), indicating that differences in phagocytosis susceptibility were not due to differences in growth.

**Figure 2:**
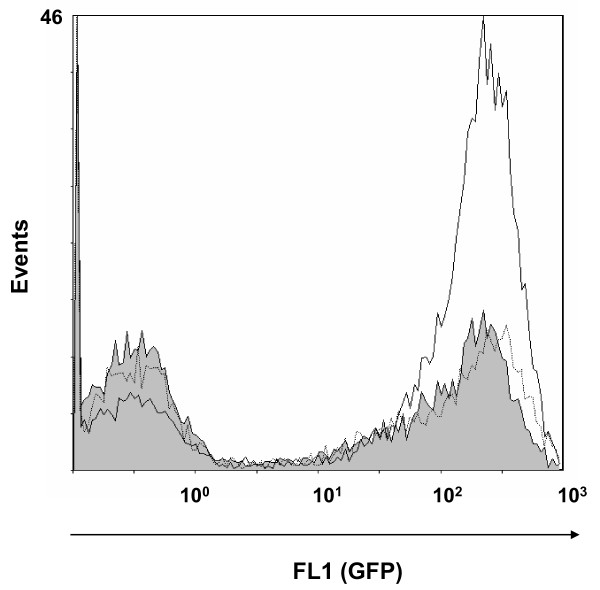
**VtaA8 and VtaA9 did not reduce bacterial attachment to PAM.** Flow cytometry of PAM after 1 h incubation at 0°C with EPI300 (pACYC184 pEGFP) (gray histogram), EPI300 (pMCH-vtaA8 pEGFP) (solid line) and EPI300 (pMCH-vtaA9 pEGFP) (dotted line).

Attachment of the clones was also examined by incubating the clones with PAM on ice. EPI300 (pMCH-vtaA8 pEGFP) and EPI300 (pMCH-vtaA9 pEGFP) showed no reduction in adhesion to the surface of the PAM, as compared to EPI300 (pACYC184 pEGFP) (Figure 2), indicating that the differences observed in phagocytosis assays were not due to differences in adhesion ability. In fact, a slight increase in attachment was observed in EPI300 (pMCH-vtaA8 pEGFP).

### Bacterial survival in the presence of PAM

Survival kinetics of EPI300 (pMCH-vtaA8) and EPI300 (pMCH-vtaA9) was examined after 1 h, 2 h, 3 h and 5 h of incubation with PAM. Although EPI300 (pMCH-vtaA8) showed better survival after 1 h of incubation and EPI300 (pMCH-vtaA9) after 2 h or longer incubation times, no significant differences with EPI300 (pACYC184) were observed. This may indicate that there is a small difference in survival (not detected by this test) that can be discerned only after successive passes with PAM (as the selection of the clones was performed).

### Surface detection of VtaA8 and VtaA9

**Figure 3:**
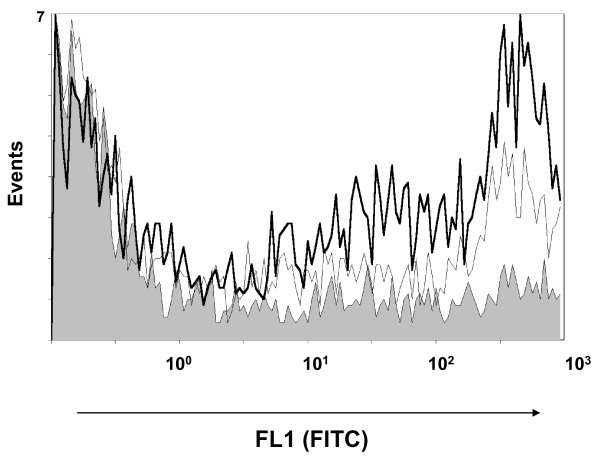
**Detection of VtaA8 and VtaA9 on the surface of the corresponding clone.*** E. coli * EPI300 (pMCH-vtaA8) (bold line) and EPI300 (pMCH-vtaA9) (fine line) were incubated with monoclonal antibody 69C6 directed against VtaA8 and VtaA9. Reaction was then detected with an anti-mouse antibody conjugated with FITC and analyzed by flow cytometry. *E. coli* EPI300 (pACYC184) served as a negative control (shown in gray).

**Figure 4:**
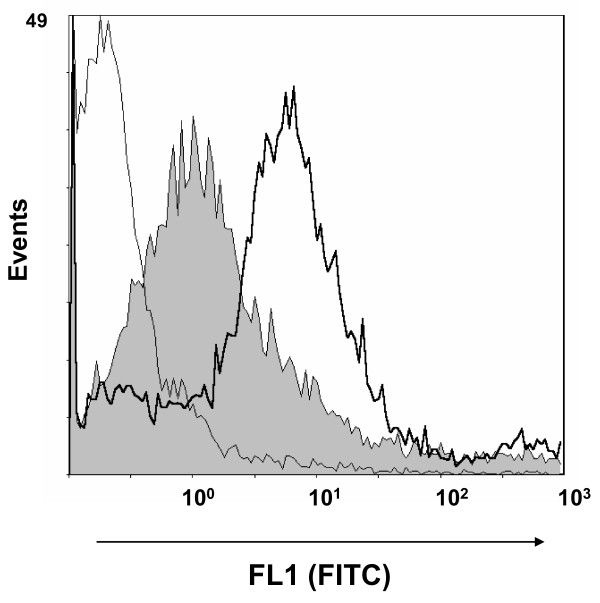
**Detection of VtaA8 and VtaA9 on the surface of* H. parasuis *.** Phagocytosis-resistant strain PC4-6P was incubated with monoclonal antibody 69C6 directed against VtaA8 and VtaA9. Reaction was detected with a FITC-conjugated anti-mouse antibody and analyzed by flow cytometry (bold line). Phagocytosis- susceptible strain F9 was included as a negative control (shown in gray). As an additional control, PC4-6P incubated only with the secondary antibody is also shown (fine line).

Preliminary observations of different auto-agglutination patterns were detected in EPI300 (pMCH-vtaA8) and EPI300 (pMCH-vtaA9) with respect to the control, pointing out differences on the surface of the clones. To confirm the surface expression of VtaA8 and VtaA9 in the clones, mAb were produced against the proteins and were used in flow cytometry. MAb 69C6, which demonstrated by ELISA and western blot reaction against both VtaA8 and VtaA9 (not shown), also showed a positive reaction with the clones EPI300 (pMCH-vtaA8) and EPI300 (pMCH-vtaA9) in flow cytometry (Figure 3). These results indicate that the proteins VtaA8 and VtaA9 are effectively expressed by the corresponding clone. In addition, mAb 69C6 also detected an epitope on the surface of strain PC4-6P, which was not detected in nasal strain F9 (Figure 4).

### Intracellular localization of phagocyted bacteria

**Figure 5:**
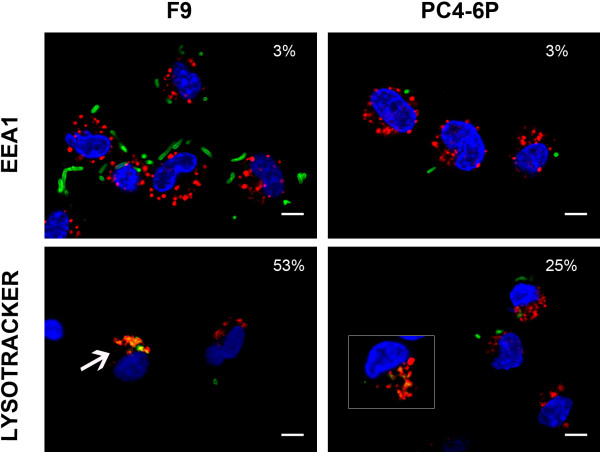
**Intracellular localization of two different* H. parasuis *strains: F9 (non-virulent strain) and PC4-6P (virulent strain) within the endosomal network of PAM.** After 1 h of incubation with PAM, bacteria were labelled with a rabbit anti-*H. parasuis* hyperimmune serum followed by an anti-rabbit-FITC (green signal) and nuclei were counterstained with DAPI (blue signal). The upper panels show the co-localization between F9 (left panel) or PC4-6P (right panel) and the early endosomal marker EEA1. EEA1 was stained with goat anti-EEA1 and anti-goat-Cy3 (red) antibodies. The lower panels show the co-localization between F9 (arrow in the left panel) or PC4-6P (detail in the inset in the right panel) and the acidic lysosomes stained with LysoTracker Red DND-99 (red signal). Percentages of co-localization are indicated in each panel. Scale bars, 5 μm. The images showing the individual fluorescences are presented in Additional file 1: Figure S1.

To explore the role of VtaA8 and VtaA9 in phagocytosis resistance, we examined the bacterial trafficking within the endosomal network. Initially, experiments with the phagocytosis-resistant strain PC4-6P and the phagocytosis-susceptible strain F9 of *H. parasuis* were performed. As previously described [20], PC4-6P, which is a systemic strain, showed a lower level of association with the macrophages than the nasal strain F9. After incubation of the *H. parasuis* strains for 1 h with PAM, we labelled EEA1 (as an early endosome marker) and the bacteria. Bacterial co-localization with this marker was quantified as the percentage of infected macrophages with co-localizing bacteria-marker. No differences were detected in the co-localization of *H. parasuis* F9 and PC4-6P with EEA1, which was observed in a low percentage of infected macrophages (Figure 5 and Additional file 1: Figure S1). Since endosomal compartments acidify during the maturation process, LysoTracker Red DND-99 was used to monitor the maturation of the endocytic compartment. With this marker, clear differences were observed in co-localization of strains PC4-6P and F9 (*p* = 0.001). Strain F9 was found in acidic compartments (co-localizing with LysoTracker Red DND-99) approximately two times more frequently than strain PC4-6P (Figure 5 and Additional file 1: Figure S1). These results suggest that F9 bacteria are properly internalized and degraded in acidic compartments while phagocytosis of the few internalized PC4-6P bacteria is postponed. 

**Figure 6:**
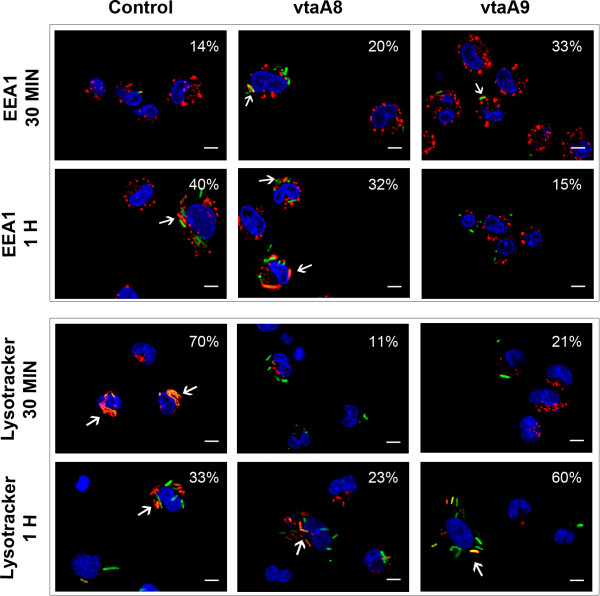
**Intracellular localization of the* E. coli *clones carrying* vtaA8 * and * vtaA9 *from* H. parasuis *within the endosomal network of PAM at different times post-infection (30 min and 1 h).*** E. coli * (pACYC184 pEGFP) (Control, first column) and the clones with *vtaA8* (pMCH-vtaA8 pEGFP; vtaA8, second column) or *vtaA9* (pMCH-vtaA9 pEGFP; vtaA9, third column) were expressing GFP (green signal). Nuclei were counterstained with DAPI (blue signal). Upper panels show the co-localization (arrows) between control, vtaA8 or vtaA9 and the early endosomal marker EEA1 (red signal). Lower panels show the co-localization (arrows) between the control, vtaA8 or vtaA9 and the acidic lysosomes stained with LysoTracker Red DND-99 (red signal). Percentages of co-localization are indicated in each panel. Scale bars, 5 μm. Individual images for each fluorescence are presented in the Additional file 2: Figure S2A and Figure S2B.

In order to examine whether VtaA8 and VtaA9 play a role in altering the endocytosis route, co-location of EPI300 (pMCH-vtaA8 pEGFP) and (pMCH-vtaA9 pEGFP) with EEA1 and lysotracker was studied. After 1 h of incubation on ice to synchronize attachment, subsequent time points (30 min and 1 h) were analyzed at 37 °C. After 30 min of phagocytosis, a small percentage of macrophages showed the control EPI300 (pACYC184 pEGFP) in EEA1-positive compartments, while the majority had the *E. coli* control in lysotracker-positive compartments, indicating its main localization in acidic compartments (Figure 6, Additional file 2: Figure S2A and Figure S2B). In contrast, EPI300 (pMCH-vtaA8 pEGFP) and EPI 300 (pMCH-vtaA9 pEGFP) were detected in EEA1-positive compartments more frequently than the control (20% and 33% respectively vs. 14%) at 30 min, but these differences were not statistically significant (*p* > 0.05). In addition, EPI300 (pMCH-vtaA8 pEGFP) and EPI300 (pMCH-vtaA9 pEGFP) showed a reduced co-localization with lysotracker after 30 min of phagocytosis as compared to the control (11% and 21% vs. 70%, *p* < 0.001; Figure 6, Additional file 2: Figure S2A and Figure S2B).

After 1 h of phagocytosis, the percentage of macrophages with EPI300 (pACYC184 pGFP) in EEA1-positive compartments increased as compared to 30 min (although not statistically significative), while the co-localization with the lysotracker showed a reduction (*p* = 0.02; Figure 6, Additional file 2: Figure S2A and Figure S2B). These results are compatible with a second wave of phagocytosis of these bacteria. Co-localization of EPI300 (pMCH-vtaA8 pEGFP) with EEA1 and lysotracker was found in a higher percentage of macrophages after 1 h than at 30 min, indicating a progressive trafficking within the endocytic pathway. This progression was more clearly observed in EPI300 (pMCH-vtaA9 pEGFP) infected macrophages, which showed a high percentage of co-localization with lysotracker after 1 h of phagocytosis (*p* = 0.004; Figure 6, Additional file 2: Figure S2A and Figure S2B).

Taken together, these results support a delay in the processing by macrophages of the *E. coli* clones carrying the VtaA8 or VtaA9 genes with respect to the control carrying the empty vector.

No apoptosis, as determined by caspase-3 detection, was observed after incubation of PAM with *H. parasuis* strains PC4-6P or F9, or after incubation with the *vtaA8* and *vtaA9* clones (not shown).

### Opsonophagocitosis

**Figure 7:**
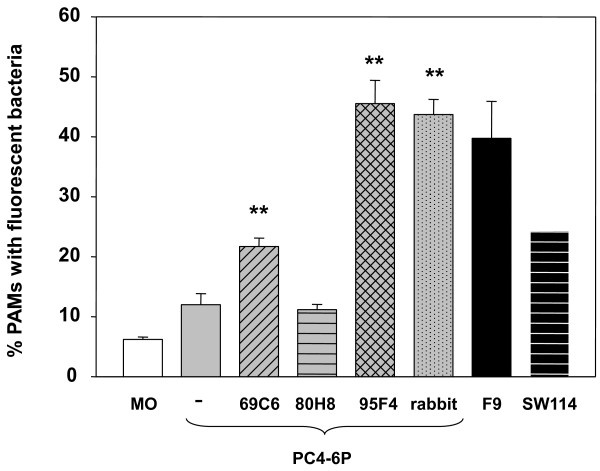
**Opsonic capacity of monoclonal antibodies anti-Vta8 and VtaA9 as determined in opsophagocytosis assay.** Phagocytosis-resistant strain PC4-6P (gray bars) was incubated with monoclonal antibodies 69C6, 95F4 and 80H8, against VtaA8 and VtaA9, to allow opsonization of the bacteria. The opsonic capacity of these antibodies was then evaluated in an opsonophagocytosis assay with porcine alveolar macrophages (PAM). Opsonization with a rabbit anti-*H. parasuis* serum was included as a positive control. Also, two phagocytosis-susceptible strains, SW114 and F9 (black bars), were included in the assay. MO indicates non-infected macrophages (white bar). The results show the mean of the percentage of PAM with associated bacteria from triplicate wells, and the error bars are the standard deviation. For strain SW114 the error bar is too small to be seen. Significant differences were detected between the phagocytosis after PC4-6P opsonization with 69C6, 95F4 and rabbit anti-*H. parasuis* compared to the non-opsonized PC4-6P (Student *t*-test, *p* < 0.001). Monoclonal 80H8 demonstrated no opsonic capacity.

The opsonic capacity of mAb 69C6 (isotype IgG2b) and 95F4 (selected for its reaction against VtaA8 and VtaA9 in ELISA, but not in western blotting; IgM) was evaluated. After incubation of the phagocytosis-resistant strain PC4-6P with the antibodies to allow for opsonization, phagocytosis was examined by flow cytometry. Opsonization of the resistant strain PC4-6P with mAb 69C6 or 95F4 yielded the strain susceptible to the phagocytosis by PAM, to levels similar to the nasal strains included in the assay (Figure 7). MAb 80H8 (isotype IgG2b) showed no opsonic capacity in this assay (Figure 7).

## Discussion

In this study, we provide evidence showing that two trimeric autotransporters of *H. parasuis*, VtaA8 and VtaA9, are surface-exposed proteins that are involved in resistance to phagocytosis. Since the production of mutants in *H. parasuis* is hindered by low and strain-dependent transformation efficiency [7,28], we decided to use the strategy of studying the function of specific genes in the heterologous host, *E. coli*. Thus, the expression of the individual proteins in *E. coli*, although not enough to prevent phagocytosis, produced a delay in the phagocytosis process. This strategy allowed us also to circumvent the problem derived from the existence of several *vtaA* in *H. parasuis*[9], which suggests a functional redundancy.

Trimeric autotransporters mediate virulence mechanisms in other Gram-negative bacteria [29-31] and their involvement in phagocytosis resistance has been previously demonstrated in *Neisseria meningitiditis*, a Gram-negative bacterium capable of producing systemic disease [32].

Recently, VtaA genes of group 1 (in which *vtaA8* and *vtaA9* are included) have been found widely represented in virulent strains of *H. parasuis*[9,33]. When expressed in *E.coli*, VtaA8 and VtaA9 promoted resistance to phagocytosis by PAM in an attachment-independent way. Both, flow cytometry and fluorescent microscopy support the role of these proteins in phagocytosis, but the proteins were unable to block the process and the bacteria eventually proceeded into the endocytic network. Our results indicate that VtaA8 and VtaA9 alone are not sufficient to avoid phagocytosis, and other factors may be involved, such as capsules, as previously suggested [20].

The N-terminal domain of trimeric autotransporters is exposed to the external media and to the host immune response [11]. The exposure of VtaA8 and VtaA9 on the bacterial surface was demonstrated with monoclonal antibodies. The presence of these proteins on the surface of virulent strain PC4-6P, while absent on the non-virulent strain F9, support the association of these trimeric autotransporters with virulence. In addition, the antibodies produced against the VtaA8 and VtaA9 of the Nagassaki strain were able to recognize the heterologous virulent strain PC4-6P. Although a mixture of VtaA was not able to fully protect against a severe *H. parasuis* challenge [34], this cross-reactivity supports the potential of these proteins as candidates in vaccine formulations against *H. parasuis*. VtaA8 and VtaA9 probably are redundant proteins affecting the PAM through the same mechanism, since co-incubation with both proteins (both clones) did not synergistically increase the effect against bacterial phagocytosis. Directing the immune response to common epitopes from both proteins can constitute a rational to improve the probability of vaccine success. Nevertheless, the choice of the adjuvant is also important for the induction of the right antibody subclass, since not all the IgG isotypes have the same capacity to promote opsonophagocytosis [35,36].

In conclusion, VtaA8 and VtaA9 are surface exposed proteins that play a role in phagocytosis resistance in virulent strains of *H. parasuis*. VtaA8 and VtaA9 share epitopes, which are also present on the surface of heterologous virulent strains. These properties make these proteins promising vaccine candidates against Glässer’s disease.

## Competing interests

None of the authors of this paper has a financial or personal relationship with people or organisations that could inappropriately influence or bias the content of this study.

## Authors’ contributions

MCH carried out the genomic library screening, phagocytosis assays, fluorescence microscopy, production and characterization of monoclonal antibodies and drafted the manuscript. MB participated in the fluorescence microscopy assays. NGM carried out the construction of the genomic library and participated in the production and characterization of the monoclonal antibodies. AD participated in the production of monoclonal antibodies. VA conceived the study and its design and coordinated it, participated in phagocytosis experiments and helped to draft the manuscript. All authors read and approved the final manuscript.

## Supplementary Material

Additional file 1 **Figure S1. Intracellular co-localization of two different* H. parasuis *strains within the endosomal network of PAM.** Nucleus counterstained with DAPI is shown in blue (first panel); bacteria (F9 or PC4-6P) labelled with FITC are visualized in green (second panel); detection of early endosomal marker (EEA1) or acidic lysosomes (Lysotracker) is shown in red (third panel); merged images are shown in the last panel. Detail of the co-localization between PC4-6P and Lysotracker is shown in the inset. Scale bars, 5 μm. Click here for file

Additional file 2 **Figure S2. A. Intracellular co-localization of EPI300-pACYC184 pEGFP (Control), EPI300-pMCH-vtaA8 pEGFP (vtaA8) and EPI300-pMCH-vtaA9pEGFP (vtaA9) bacteria with early endosomal antigen 1 (EEA1) at different times post-infection (30 min and 1 h).** Nucleus counterstained with DAPI is shown in blue (first panel); bacteria (Control, vtaA8 and vtaA9) expressing GFP are visualized in green (second panel); detection of EEA1 is shown in red (third panel); merged images are shown in the last panel. Scale bars, 5 μm. B. Intracellular localization of EPI300-pACYC184 pEGFP (Control), EPI300-pMCH-vtaA8 pEGFP (vtaA8) and EPI300-pMCH-vtaA9pEGFP (vtaA9) bacteria in acidic compartments at different times post-infection (30 min and 1 h). Nucleus counterstained with DAPI is shown in blue (first panel); bacteria (Control, vtaA8 and vtaA9) expressing GFP are visualized in green (second panel); detection of acidic lysosomes (as detected with Lysotracker) is shown in red (third panel); merged images are shown in the last panel. Scale bars, 5 μm. Click here for file
