# Supplementary figures and images for: VtaA8 and VtaA9 from Haemophilus parasuis delay phagocytosis by alveolar macrophages
Source: Vet Res. 2012 Jul 27;43(1):57. doi: 10.1186/1297-9716-43-57 (PMC3462726; doi:10.1186/1297-9716-43-57)

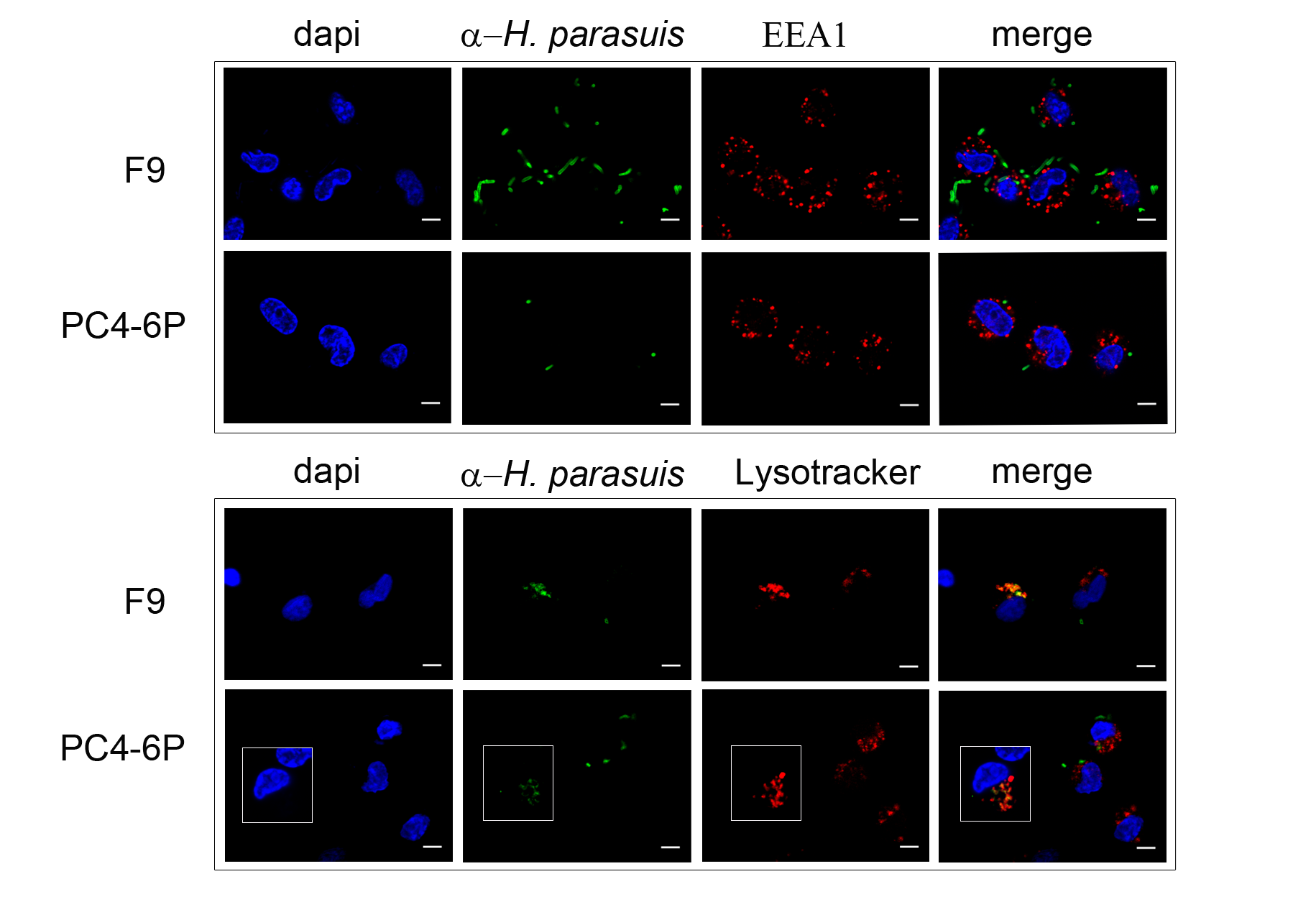

Supplement: Additional file 1 — Figure S1. Intracellular co-localization of two different H. parasuis strains within the endosomal network of PAM. Nucleus counterstained with DAPI is shown in blue (first panel); bacteria (F9 or PC4-6P) labelled with FITC are visualized in green (second panel); detection of early endosomal marker (EEA1) or acidic lysosomes (Lysotracker) is shown in red (third panel); merged images are shown in the last panel. Detail of the co-localization between PC4-6P and Lysotracker is shown in the inset. Scale bars, 5 μm. [file 1297-9716-43-57-S1.tiff]

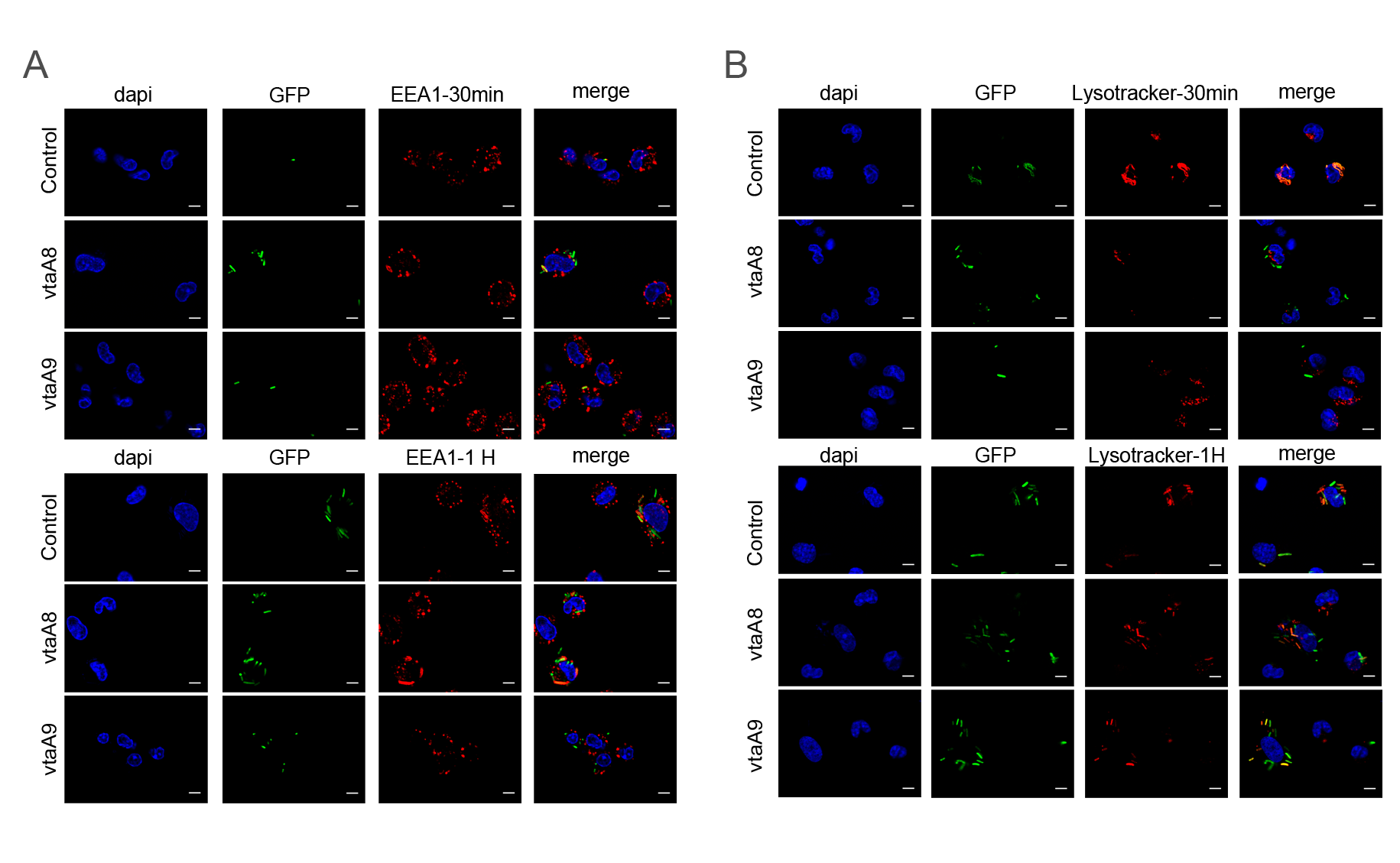

Supplement: Additional file 2 — Figure S2. A. Intracellular co-localization of EPI300-pACYC184 pEGFP (Control), EPI300-pMCH-vtaA8 pEGFP (vtaA8) and EPI300-pMCH-vtaA9pEGFP (vtaA9) bacteria with early endosomal antigen 1 (EEA1) at different times post-infection (30 min and 1 h). Nucleus counterstained with DAPI is shown in blue (first panel); bacteria (Control, vtaA8 and vtaA9) expressing GFP are visualized in green (second panel); detection of EEA1 is shown in red (third panel); merged images are shown in the last panel. Scale bars, 5 μm. B. Intracellular localization of EPI300-pACYC184 pEGFP (Control), EPI300-pMCH-vtaA8 pEGFP (vtaA8) and EPI300-pMCH-vtaA9pEGFP (vtaA9) bacteria in acidic compartments at different times post-infection (30 min and 1 h). Nucleus counterstained with DAPI is shown in blue (first panel); bacteria (Control, vtaA8 and vtaA9) expressing GFP are visualized in green (second panel); detection of acidic lysosomes (as detected with Lysotracker) is shown in red (third panel); merged images are shown in the last panel. Scale bars, 5 μm. [file 1297-9716-43-57-S2.tiff]
